# Improving the Quality of Voluntary Medical Male Circumcision through Use of the Continuous Quality Improvement Approach: A Pilot in 30 PEPFAR-Supported Sites in Uganda

**DOI:** 10.1371/journal.pone.0133369

**Published:** 2015-07-24

**Authors:** John Byabagambi, Pamela Marks, Humphrey Megere, Esther Karamagi, Sarah Byakika, Alex Opio, Jacqueline Calnan, Emmanuel Njeuhmeli

**Affiliations:** 1 USAID Applying Science to Strengthen and Improve Systems (ASSIST) Project, University Research Co., LLC (URC), Kampala, Uganda; 2 USAID Applying Science to Strengthen and Improve Systems (ASSIST) Project, University Research Co., LLC (URC), Bethesda, Maryland, United States of America; 3 Ministry of Health, Kampala, Uganda; 4 Health Team, United States Agency for International Development (USAID), Kampala, Uganda; 5 Office of HIV/AIDS, Global Health Bureau, United States Agency for International Development (USAID), Washington, District of Columbia, United States of America; Asociacion Civil Impacta Salud y Educacion, PERU

## Abstract

**Background:**

Uganda adopted voluntary medical male circumcision (VMMC) (also called Safe Male Circumcision in Uganda), as part of its HIV prevention strategy in 2010. Since then, the Ministry of Health (MOH) has implemented VMMC mostly with support from the United States President’s Emergency Plan for AIDS Relief (PEPFAR) through its partners. In 2012, two PEPFAR-led external quality assessments evaluated compliance of service delivery sites with minimum quality standards. Quality gaps were identified, including lack of standardized forms or registers, lack of documentation of client consent, poor preparedness for emergencies and use of untrained service providers. In response, PEPFAR, through a USAID-supported technical assistance project, provided support in quality improvement to the MOH and implementing partners to improve quality and safety in VMMC services and build capacity of MOH staff to continuously improve VMMC service quality.

**Methods and Findings:**

Sites were supported to identify barriers in achieving national standards, identify possible solutions to overcome the barriers and carry out improvement plans to test these changes, while collecting performance data to objectively measure whether they had bridged gaps. A 53-indicator quality assessment tool was used by teams as a management tool to measure progress; teams also measured client-level indicators through self-assessment of client records. At baseline (February-March 2013), less than 20 percent of sites scored in the “good” range (>80%) for supplies and equipment, patient counseling and surgical procedure; by November 2013, the proportion of sites scoring “good” rose to 67 percent, 93 percent and 90 percent, respectively. Significant improvement was noted in post-operative follow-up at 48 hours, sexually transmitted infection assessment, informed consent and use of local anesthesia but not rate of adverse events.

**Conclusion:**

Public sector providers can be engaged to address the quality of VMMC using a continuous quality improvement approach.

## Introduction

In 2007, World Health Organization (WHO) and United Nations Programme on AIDS (UNAIDS) recommended that voluntary medical male circumcision (VMMC) (also called Safe Male Circumcision in Uganda) be considered as part of a comprehensive package for prevention of HIV in countries with high HIV prevalence and low rates of circumcision [1]. Uganda is one of these countries, with an estimated HIV prevalence rate of 7.3 percent in 2011, up from 6.4 percent in 2004 [2]; and prevalence of circumcision at 26 percent [3]. It is estimated that VMMC will avert 3.36 million and more than 300,000 new HIV infections in the 13 focus countries and Uganda, respectively, between 2011 and 2025 with scale-up of VMMC services [4]. However, to benefit from this proven intervention, it requires that it is offered on a large scale and must reach up to 80 percent of the target population of men aged 15 to 49 years old [4]. The number of men who needed to be circumcised in Uganda was estimated to be around 4.3 million, in order to achieve such meaningful impact with VMMC [4].

Uganda adopted VMMC as part of its HIV prevention strategy in 2010. The Uganda Ministry of Health (MOH) developed a VMMC policy [5] and issued minimum standards to guide national VMMC practice [6]. The MOH has been implementing VMMC mostly with support from the United States President’s Emergency Plan for AIDS Relief (PEPFAR) through USAID, the CDC and the Department of Defense (DOD), using various implementing partners spread across the country. As a new intervention that was undergoing rapid scale-up, it is important that high standards of quality be maintained—more so since VMMC is a surgical intervention and has been noted previously that rapid scale-up may compromise the quality of service [7–9]. Concerns about decrease of quality of services with rapid scale-up have also been demonstrated in South Africa [9]. However, Zimbabwe was able to scale up while at the same time improving service quality, probably due to a strong Quality Assurance/Quality Improvement (QA/QI) component, training of providers and the MOH-designed program [8].

Prior to 2012, little was known about the quality of VMMC in Uganda. It was not until 2012 that two external quality assessments (EQA) were conducted by an interagency PEPFAR team to evaluate compliance of service delivery sites with national and international minimum quality standards for VMMC. Serious quality gaps were identified, including lack of standardized client record forms or registers, circumcision of clients with no evidence of consent, procedures done by providers not properly trained and use of general anesthesia for routine VMMC procedures. Additionally, many sites were missing necessary equipment and supplies for conducting surgeries and management of emergencies and were not screening clients for sexually transmitted infections (STI) or treating clients diagnosed with STIs. Sites also had no documentation of linking those who tested positive for HIV with other services, like antiretroviral clinics. Among the many recommendations emerging from the EQA was the need for health facilities and implementing partners (IPs) to urgently institute continuous quality improvement (CQI) interventions to address the quality gaps including developing standardized client record forms [10].

In early 2013, USAID—through USAID Applying Science to Strengthen and Improve Systems (USAID ASSIST) Project—provided technical support in quality improvement to the MOH and all 10 USAID- and DOD-funded IPs supporting VMMC services in Uganda. The objectives were to improve the quality and safety of VMMC services as recommended by the EQA team and build capacity of District Health Offices (DHO), MOH AIDS Control Program (ACP), MOH Quality Assurance Department (QAD), IPs and providers to making ongoing improvements.

Since January 2013, USAID ASSIST has gained important insights about addressing gaps in VMMC quality. This article explains lessons learned so that they may be replicated in other priority countries facing similar quality gaps and challenges in their VMMC programs, since most of the previous work on VMMC quality has focused on the availability of minimum service components [8], documenting rates of adverse events [7,11,12] and adoption of surgical efficiency models [13]. Lessons learned from this activity may also be used to address quality gaps in other health programs. We describe the steps taken to begin the process, how tools were developed and used to assess quality, how we supported facilities to address identified gaps and the results achieved.

## Materials and Methods

Data reported below for performance of the 30 sites on the 53 VMMC quality standards and on client-level indicators were collected and self-reported by health facility staff based on their review of registers and patient records. Data by site were aggregated by facility staff and shared without patient identification with USAID ASSIST staff. None of the authors of this paper had access to individual patient information or identifiers.

### Improvement Approach

Our approach applied CQI in a collaborative improvement structure, organizing teams of providers to conduct site-level tests of change in care processes through repeated application of a Plan-Do-Study-Act (PDSA) cycle and linking their efforts through deliberate mechanisms for sharing learning across all sites [14,15]. An improvement collaborative is a time-limited, organized improvement effort by a network of team focused on common aims, making changes in support systems and delivery processes to achieve better results, and shared learning among the teams to spread successful changes to all teams [16–19]. We organized a collaborative involving 29 fixed and one mobile (Table 1) VMMC delivery sites located in 26 districts to facilitate rapid dissemination of successful changes to improve VMMC quality among the 30 teams. The 30 sites comprising the collaborative were selected by the 10 PEPFAR-funded IPs; each IP selected three of its sites to participate in the collaborative. USAID ASSIST recommended that the IPs select high-volume sites and/or sites with known quality problems.

**Table 1:** The level and ownership of USAID ASSIST-supported sites.

Level	Public	Military/Police	Private not for profit	Total
Regional Referral Hospital	3	0	0	3
General Hospital	7	4	4	15
Health Center IV	8	0	0	8
Health Center III	0	2	0	2
Heath Center II	0	0	1	1
Mobile Vans	0	0	1	1
TOTAL	18	6	6	30

Throughout 2013, USAID ASSIST provided intensive (at least once-a-month site visits) support to the 30 sites, DHOs and IPs to identify barriers in achieving national and international VMMC standards, and to develop and implement improvement plans to overcome these barriers. The intensive support included monthly onsite coaching using a continuous quality improvement approach. This CQI approach involved supporting teams in using available data collected at the site level to identify gaps in service delivery and guiding the team to identify possible solutions (*changes*). The possible solutions (*changes*) were then tested through an iterative process also called Plan, Do, Study, Act (PDSA) cycles [15,17], in which teams made changes to service delivery processes to address a gap on small scale and then continued to expand and refine the changes while collecting performance data to objectively measure whether they had bridged the gap. Once changes had been determined to yield improvement in the small-scale tests, they were scaled up in the clinic. All teams participating in the collaborative were brought together in quarterly peer-to-peer learning sessions in which they shared experiences and further refined their intervention strategies based on learning from other teams. In this way, teams that still had poor results were supported to refine their tests based on learning from teams with better results. Once a specific change had been shown to lead to improvement, it was recommended to all teams to be made part of routine service delivery. The intervention was divided into preparatory and onsite intervention activities.

### Preparatory Meetings and Development of Standard Improvement Tools and VMMC Quality Assessment Tool

Two stakeholders meetings were convened to prepare for onsite improvement activities. The purpose of the first meeting in January 2013 was to share the implementation strategy and draft VMMC quality assessment tools. Stakeholder consultations determined that it was necessary to adapt the quality standards to the Ugandan context; some standards within the WHO toolkit were viewed to be too stringent in some aspects, for instance they required supplies and equipment, some of which were far advanced beyond those recommended by the MOH. USAID ASSIST, with technical assistance from USAID Maternal and Child Health Integrated Program (MCHIP), supported the MOH to align the WHO VMMC quality standards toolkit [20] to other existing MOH standards, policies and guidelines.

The assessment tool developed for Uganda encompassed 53 standards, organized into seven areas, each with a subset of standards (Table 2): (a) *Management Systems* with 10 standards, (b) *Supplies*, *Equipment and Environment* with 6 standards, (c) *Registration*, *Group Education and Information Education and Communication* with 4 standards, (d) *Individual Counseling and HIV Testing for VMMC Clients* with 6 standards, (e) *Male Circumcision Surgical Procedure* with 10 standards, (f) *Monitoring and Evaluation* with 4 standards and (g) *Infection prevention* with 13 standards.

**Table 2:** Ministry of Health VMMC quality standards.

Area	Standards
1. Management Systems (10 standards)	1. Relevant SMC policies, guidelines and standards are available and staff are aware of them.
	2. The site has a written service delivery plan (minimum 1 year plan).
	3. The SMC clinic is able to meet demand for services.
	4. The SMC clinic or facility has clearly defined staff roles and responsibilities.
	5. The SMC clinic or facility has the human resources available according to the SMC service delivery plan.
	6. Staff receives mentoring and support.
	7. Client flow chart is available in the facility.
	8. Service delivery data are used for planning and improvement of service delivery.
	9. Moderate and severe adverse events or complications are reviewed.
	10. The facility / SMC clinic has a functional supply and equipment ordering system.
2. Supplies, Equipment and Environment (6 standards)	1. The physical facilities are appropriate for SMC service provision.
	2. The necessary equipment is available for performing SMC surgeries.
	3. The necessary commodities are available for performing surgeries.
	4. Adequate supplies of medicines and commodities (HIV test kits, condoms) are available for non-surgical aspects of SMC service provision.
	5. An emergency resuscitation system exists and medications / supplies are available with immediate access.
	6. Adequate measures are in place for managing moderate to severe complications and adverse events.
3. Registration, Group Education and IEC (4 standards)	1. The client is correctly recorded in the register and given a client ID.
	2. The facility has appropriate information and educational materials on SMC and other reproductive health.
	3. Group education delivered with correct information.
	4. Group education delivered with appropriate techniques.
4. Individual Counseling and HIV Testing for SMC Clients (6 standards)	1. The provider provides appropriate individual counseling on SMC.
	2. The provider provides routine HIV testing for every client.
	3. The provider is properly giving results and post-test counseling.
	4. The provider uses appropriate counseling skills throughout the session.
	5. All clients receive condoms along with appropriate counseling and instructions on their use.
	6. The provider obtains informed consent from clients.
5. Male Circumcision Surgical Procedure (10 standards)	1. The provider correctly takes history.
	2. The provider correctly performs pre-operation examination.
	3. The provider prepares the client for surgery.
	4. The provider administers anesthetic and performs dorsal slit correctly.
	5. The provider achieves hemostasis, sutures the wound and applies the dressing correctly.
	6. Provider is able to respond appropriately to an emergency situation.
	7. The provider completes the procedure and assists the client to the post-operative area.
	8. The provider monitors immediate post-op client.
	9. The provider gives client appropriate post-op care instructions.
	10. Client records are updated and completed prior to discharge.
	11. The provider correctly manages initial follow-up.
6. Monitoring and Evaluation (4 standards)	1. Availability of relevant tools in SMC clinic.
	2. Data are correctly transferred from SMC Client Record to the SMC Client Counseling, Testing and Follow-up Register.
	3. Client records are complete and correspond with the SMC Client Counseling, Testing and Follow-up Register.
	4. Data are correctly summarized, reported and filed.
7. Infection Prevention (13 standards)	1. The concentration and use of antiseptics are according to the standards.
	2. The process of cleaning rooms between and after procedures is performed according to the standards.
	3. The preparation of a disinfectant cleaning solution is performed according to the standards.
	4. The cleaning equipment is decontaminated, cleaned and dried before reuse or storage according to the standards.
	5. The decontamination of instruments and other articles (immediately after use and before cleaning) is performed according to the standards.
	6. The process of cleaning instruments and other items is performed according to the standards.
	7. The process of packaging of items to be sterilized is performed according to the standards.
	8. The process sterilization is performed according to the standards.
	9. The storage process of sterile or high-level disinfected items is performed according to the standards.
	10. Waste is disposed of/handled appropriately.
	11. The system for interim storage is appropriate.
	12. The facility / SMC clinic ultimately disposes of waste properly.
	13. Disposable male circumcision kits are disposed properly.

All seven areas of the eventual assessment tool were developed based on the existing policies and guidelines of the MOH. For example, under Area 2 (Supplies, Equipment and Environment), standard four requires that medicines are available for the management of STIs; in the adapted tool, these were aligned to the national guidelines for management of STIs. The standard that requires presence of a “relevant reference tools” was changed to reflect the relevant MOH documents. Additionally, the tools were aligned to Uganda’s Health Sector Quality Improvement Framework and Strategic Plan [21]. The improvement strategy of forming site-level improvement teams followed the guidance given in the plan, and there was minimal introduction of new CQI tools. For example, the existing MOH quality improvement documentation journal was adopted.

The next round of meetings was to gain feedback on the draft tools from a wider audience. This involved a series of meetings held from January 2013 to March 2013 and field pre-testing. This activity was supported by a team of USAID MCHIP colleagues who advised the Uganda team on its adaptation of the WHO toolkit. The CQI standards in the male circumcision services quality assessment toolkit [20] was successfully adapted by the MOH and approved at the three required levels in the MOH. The first level approval was from the National Task Force (NTF) for VMMC. The second level approval was from the MOH Supervision Monitoring, Evaluation and Research technical working group. The final approval was by the MOH Senior Management Committee.

### Baseline Assessment of the Sites

USAID ASSIST requested the 10 IPs supporting VMMC services to each identify three facilities that were high volume and impacted many clients and secondly that had quality gaps to receive intensive support and act as demonstration sites for improving the quality of VMMC in Uganda. The sites actually selected were at varying levels of performance. For instance, sites from one IP who also happened to be a WHO designated VMMC training center were generally performing well on most of the quality standards and had a few gaps in client-level indicators, while other IPs included sites whose funding had been suspended due to poor service quality during the EQA.

A baseline assessment was conducted from January 2013 to March 2013 at the 30 sites using the newly developed VMMC quality standards assessment tool to identify the strengths and gaps of each site so that improvement efforts could be tailored to each site’s gaps. Assessment teams were comprised of staff from USAID ASSIST, IPs, representatives from the MOH, DHO, and staff from the health unit to minimize bias and promote ownership of the findings. At the end of every site assessment, a plan to address the identified gaps was developed with the site staff using a standardized template that indicated the following: identified gaps, proposed action(s) to address the gap(s), responsible party (i.e., the facility staff, the IP, the DHO, or USAID ASSIST) and timelines for implementing the action.

### Onsite Intervention Activities

While conducting the baseline assessment, our team noted that the majority of service providers were not skilled in implementing improvement and needed support. A three-day training in quality improvement was conducted and participants from all 30 sites, staff from the IPs and district representatives were invited. To make the training manageable, the 30 sites were divided into three groups based on geographical location. The facilitators at this training were from the MOH QAD and USAID ASSIST. Training objectives were (a) to orient VMMC service providers in CQI approaches, (b) to agree upon indicators for tracking improvement that would enable all stakeholders to track progress in a standardized way and (c) to develop action plans for initiating quality improvement activities to address the gaps identified in the baseline assessment.

After the training in improvement methods, each health facility was guided to form an improvement team based on the MOH’s National Quality Improvement Framework & Strategic Plan [21], whose purpose was to ensure improvement activities are led by the site teams to promote institutionalization and sustainability. In each site, the quality improvement team was comprised of representatives from each section within VMMC; these included circumcisers, assistant circumcisers, counsellors, data clerks and community mobilisers. Each site had one team, so in total there were 30 improvement teams from 30 health units.

A team comprised of USAID ASSIST, a representative from the DHO and the supporting IP provided onsite coaching to each intensive support site team on a monthly basis starting in April 2013, except for months when peer-to-peer learning sessions were held. Learning sessions were meetings convened quarterly by USAID ASSIST and the MOH to bring together representatives from all the intensive support site teams, district health officials and IPs to share their experiences of changes tested, including how sites had addressed cross-cutting gaps such as poor documentation.

### Measuring Improvement

Improvement was tracked by teams on performance on the 53 VMMC quality standards and performance on client-level indicators agreed upon during the training in improvement methods. A VMMC performance dashboard, a management tool that summarizes scores using color, was developed to track the performance of sites on achieving the MOH VMMC quality standards. Red signifies scores below 50 percent and indicates “poor” performance; yellow scores are 50 percent to below 80 percent and indicate “fair” performance; and green scores indicate 80 percent or more and indicate “good” performance. The dashboard was developed to summarize compliance with the 53 quality standards in a simple but explanatory manner for the site teams, district staff and IPs to make it easy to track and identify priority areas for intervention.

Performance indicators (also known as client-level indicators) were also agreed upon to track improvement of key VMMC service components. These included (a) proportion of clients with documented consent prior to circumcision, (b) proportion of clients circumcised under local anesthesia, (c) proportion of clients assessed for sexually transmitted infection before circumcision, (d) proportion of clients who experience moderate to severe adverse events after circumcision and (e) proportion of clients who return for follow-up within 48 hours, within one week and six weeks after circumcision. These data were collected on monthly basis by the QI teams and validated by the coaches at subsequent coaching visits. The validation involved crosschecking all reported data against source documents.

The data were plotted on time series charts to track progress month by month, and the results were interpreted using run chart rules [22]. In accordance with accepted rules for interpreting time series charts [22], median or mean lines are plotted for the first 10 data points in each set of aggregate data displayed and for subsequent data points in each figure. For each median or mean line, the number of times that the line graph crosses the line of central tendency is counted to indicate the number of “runs” in the time series chart. Fewer than expected number of runs for a given number of data points indicates with 95% confidence, a non-random pattern in the line graph (i.e., that there has been a change in performance due to some special cause) [22]. Six consecutive points above or below the line of central tendency indicates with 95% confidence that a shift in performance has occurred in the direction of the six points (i.e., improved performance or deteriorated performance) [22].

## Results

### Compliance with VMMC Quality Standards

A total of 30 multi-level health units (Table 1) were assessed at baseline and during follow-up coaching visits against the seven areas comprising the 53 standards. At baseline, the majority of sites that were assessed scored either fair or poor in all areas except Area 4 (Individual Counseling and HIV Testing) and Area 5 (Male Circumcision Surgical Procedure). However, in Areas 4 and 5, only seven and nine sites, respectively, contributed to the data because these areas can only be assessed when the site is in action. Results of the assessment at baseline for standards that could be assessed are shown in Table 3.

**Table 3:** Categorization of baseline findings for standards that could be assessed by category of performance.

Area of the SMC Standards	Proportion of Sites (Percentage) Categorized by Performance Category		
	Poor	Fair	Good
Management Systems	16/27 (59%)	11/27 (41%)	0/27 (0%)
Supplies, Equipment and Environment	6/29 (21%)	17/29 (59%)	6/29 (21%)
Registration, Group Education and IEC	13/27 (48%)	7/27 (26%)	7/27 (26%)
Individual Counseling and HIV Testing for VMMC Clients	1/7 (14%)	1/7 (14%)	5/7 (71%)
Male Circumcision Surgical Procedure	1/9 (11%)	2/9 (22%)	6/9 (78%)
Monitoring and Evaluation	21/29 (72%)	5/29 (17%)	3/29 (10%)
Infection Prevention	8/30 (27%)	12/30 (40%)	10/30 (33%)

**Fig 1:**
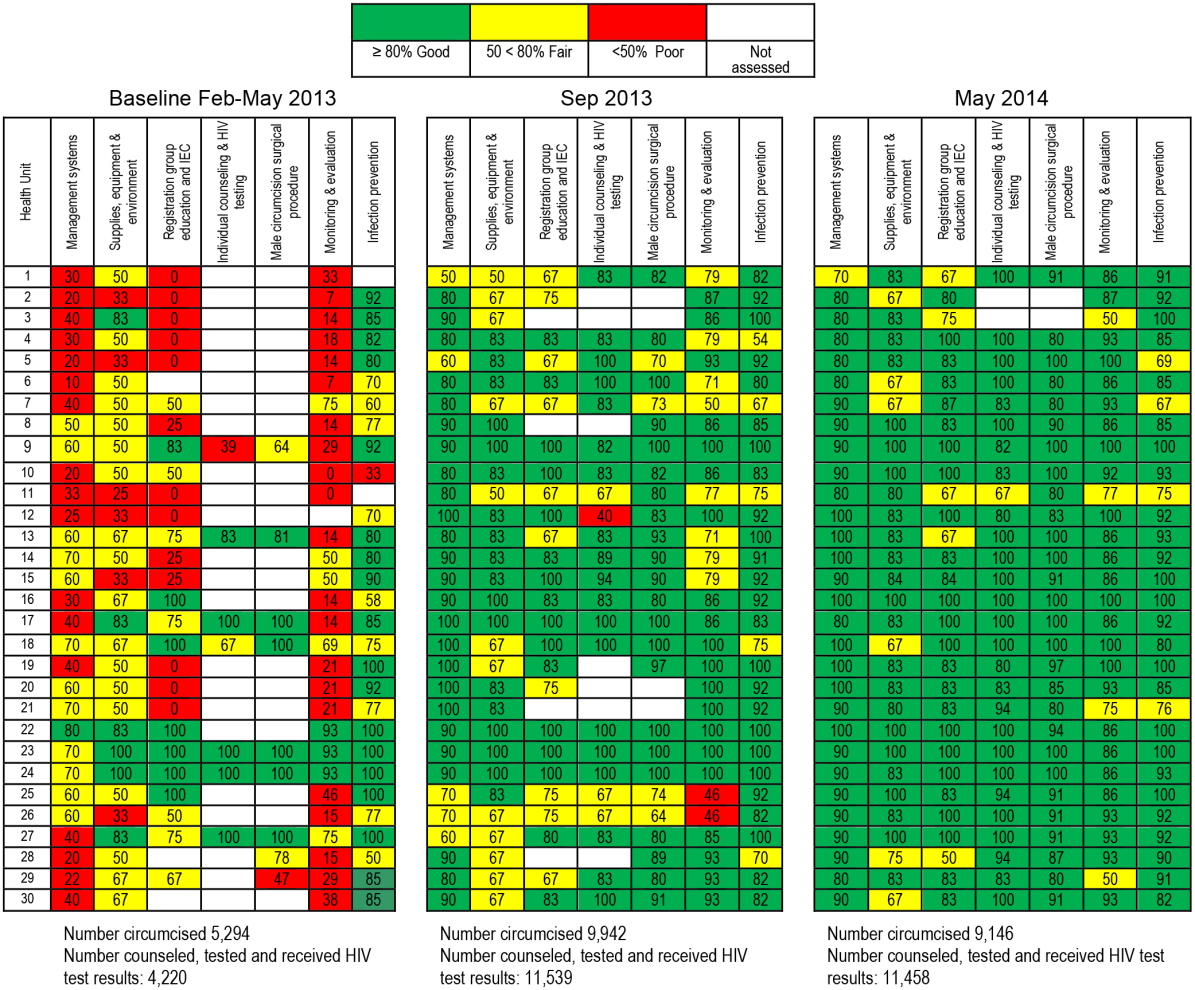
Dashboard showing the performance of intensive-support sites on seven areas of the VMMC quality standards.

**Fig 2:**
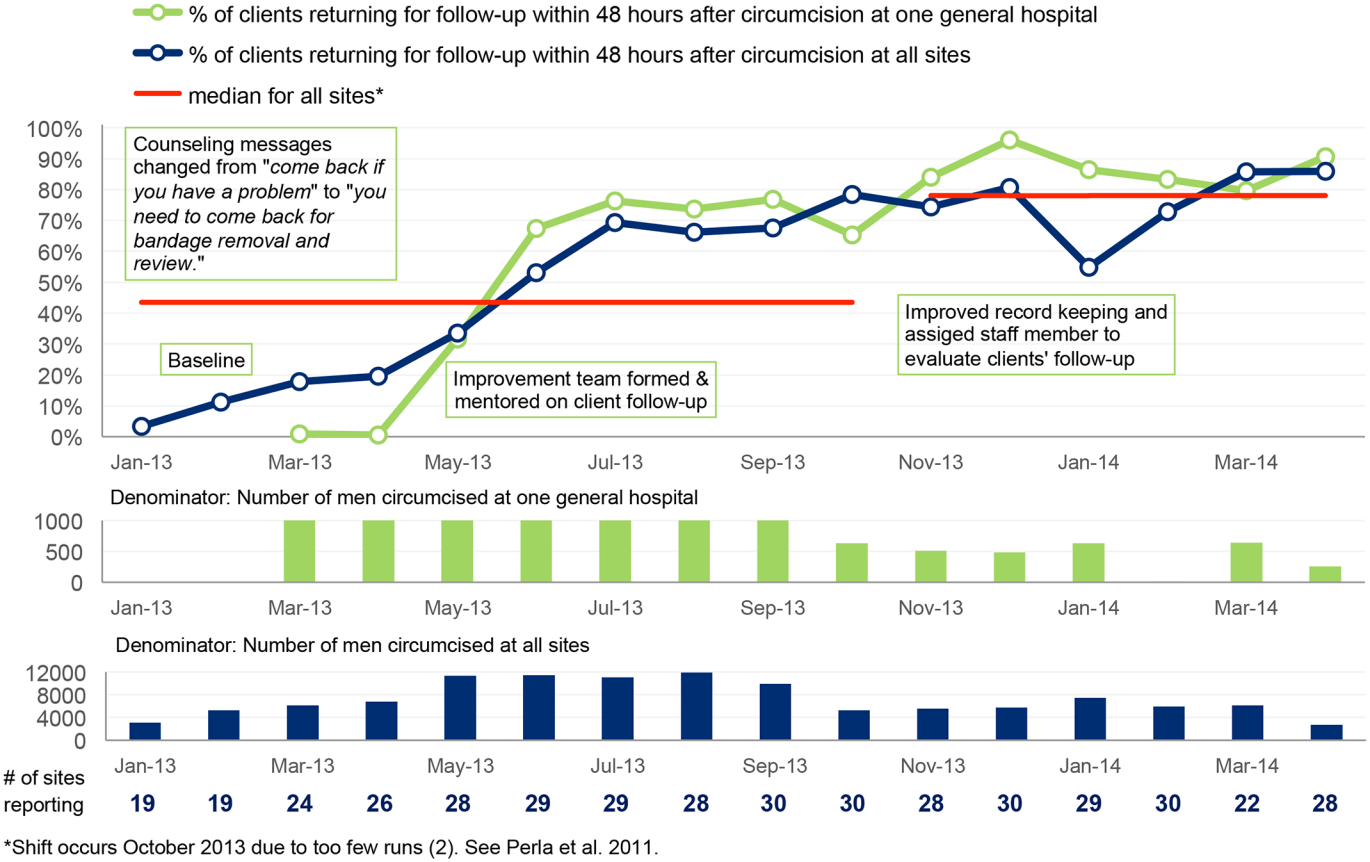
Performance of intensive-support VMMC sites on client follow-up within 48 hours after circumcision.

**Fig 3:**
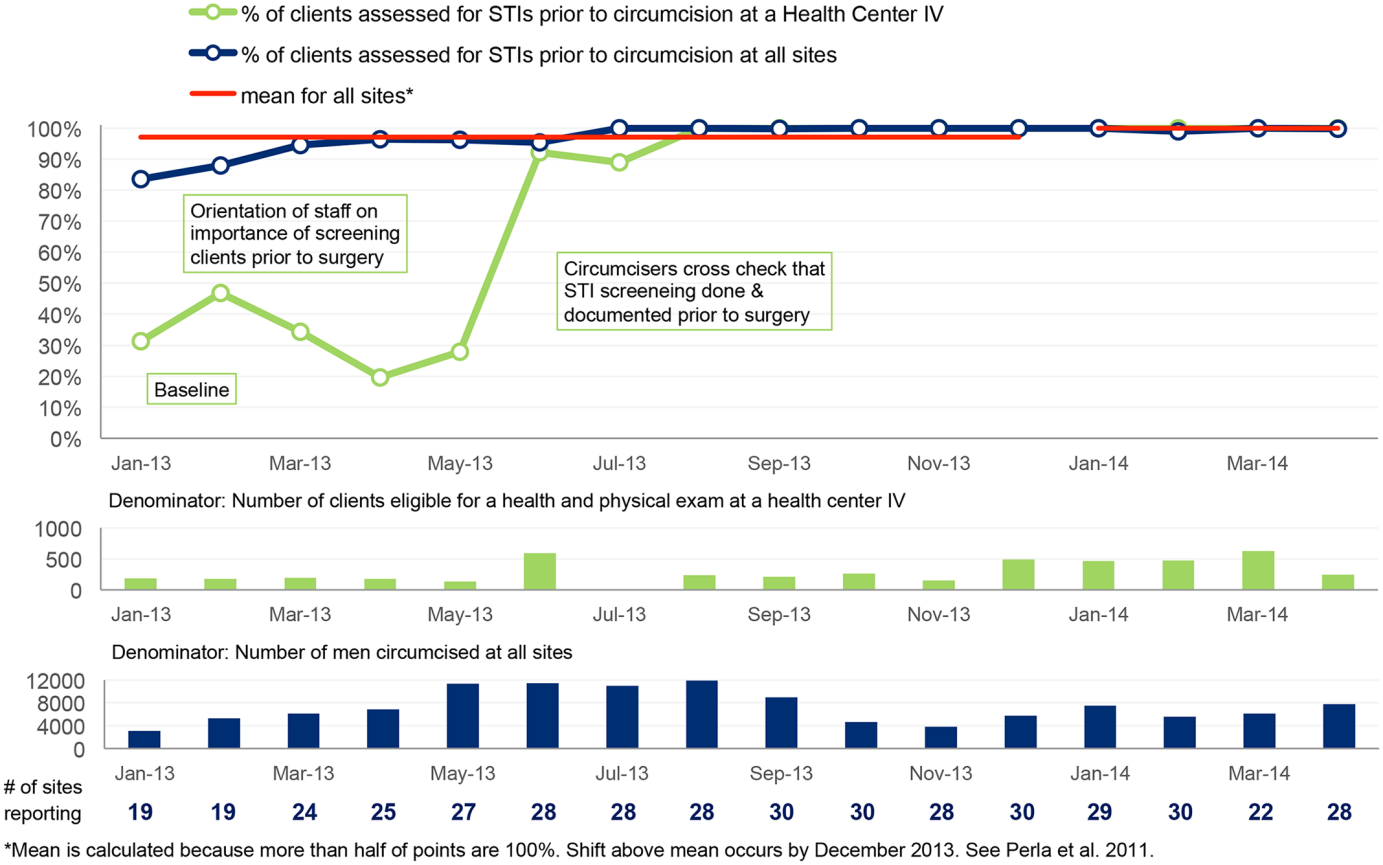
Percentage of clients assessed for STIs prior to circumcision.

**Fig 4:**
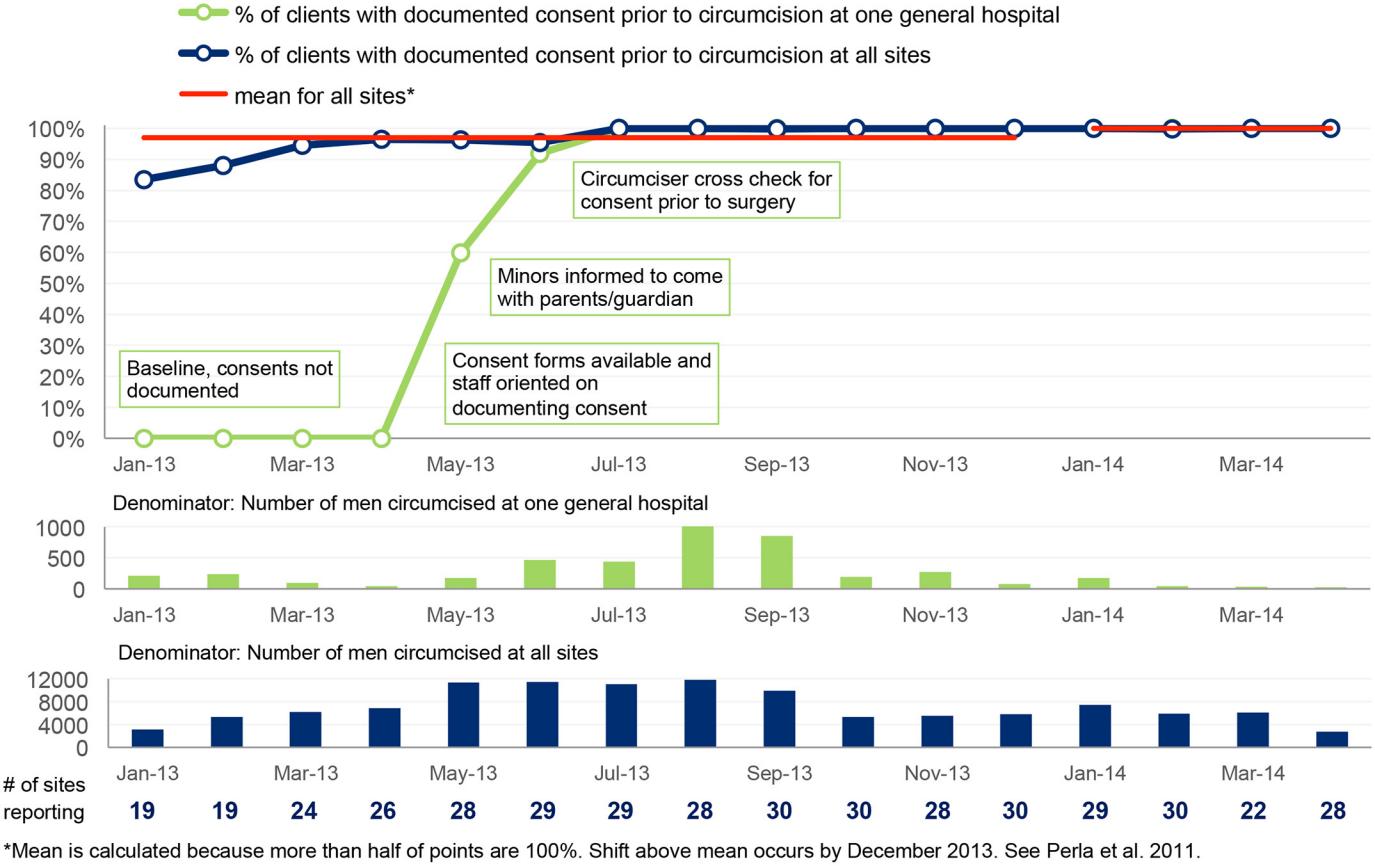
Percentage of clients with documented consent prior to circumcision.

**Fig 5:**
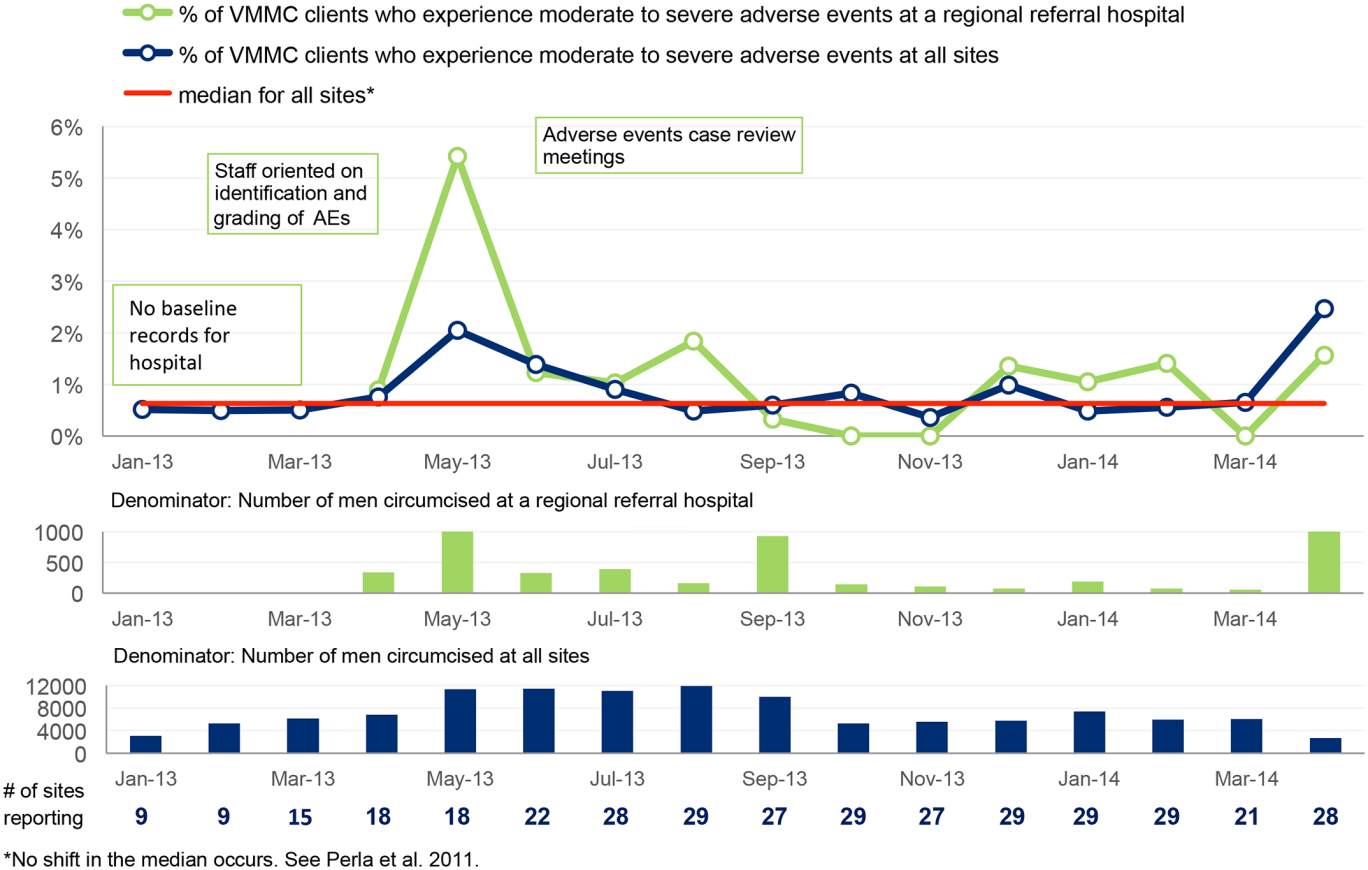
Percentage of VMMC clients who experience moderate to severe adverse events.

**Fig 6:**
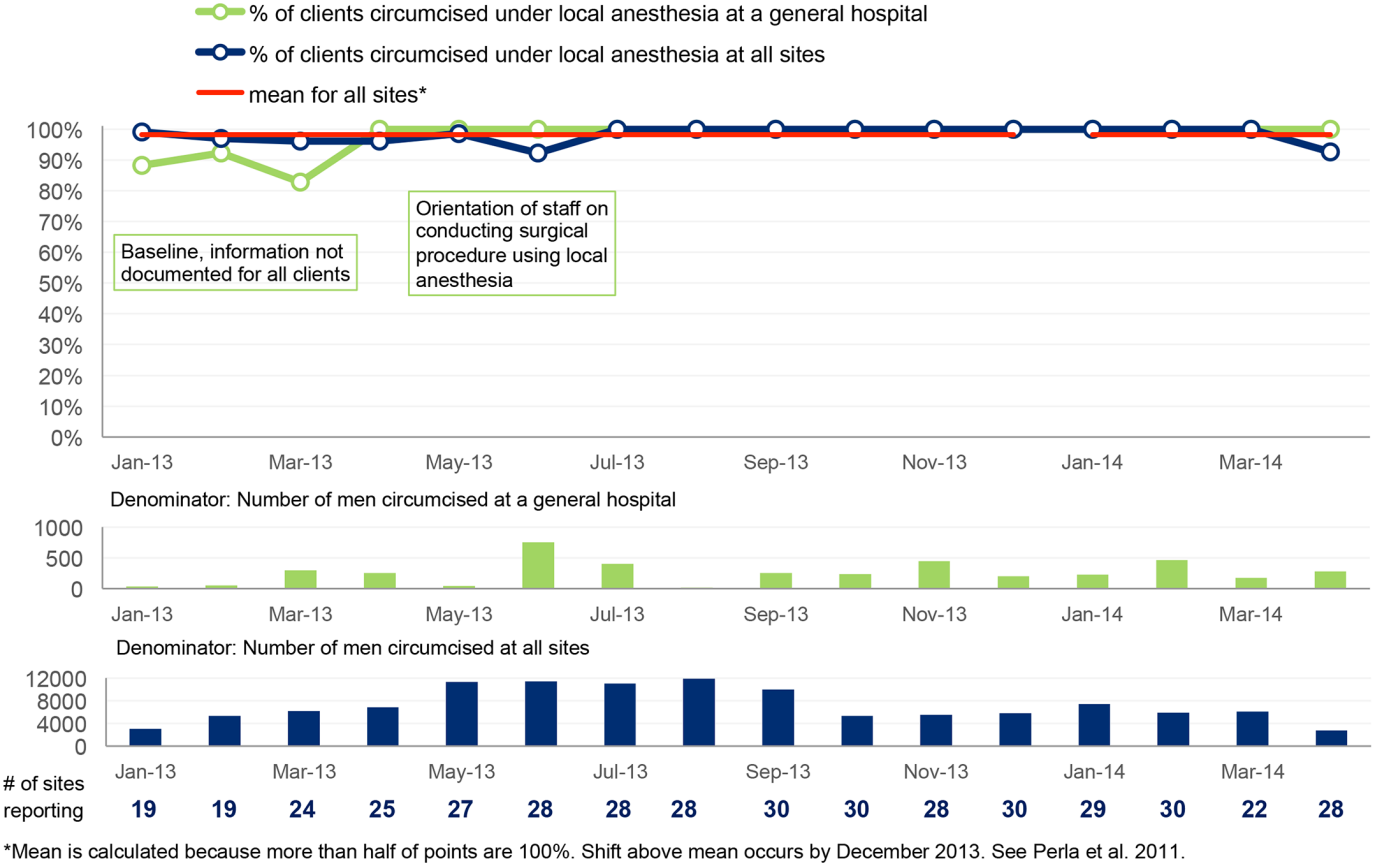
Percentage of VMMC clients who are circumcised under local anesthesia.

The baseline findings are summarized in the first set of columns in the dashboard shown in Fig 1. Site teams were supported to address gaps identified at baseline concerning the seven areas in the VMMC standards quality assessment tool. Since the baseline assessment was conducted, improvement has been observed in all areas, as shown in the columns for May 2014. A greater proportion of sites have now been assessed as performing “good” (green) in all of the seven quality areas, as shown in Fig 1. Table 4 summarizes the gaps identified by teams for some of the VMMC quality standards and the changes that teams made to close these gaps. Because individual sites introduced specific changes at different times, aggregate time series results for all 30 sites presented in Figs 2–6 are compared with results for a single site, with site-specific annotations shown to indicate when the particular site introduced each specific change. The individual sites whose results are presented in Figs 2–6 were purposefully selected to favor higher-volume sites and sites where documentation of site-specific changes was available.

**Table 4:** Gaps identified by VMMC improvement teams and changes tested to address the gaps.

Area/Standard	Identified Gaps	Changes to Address Gaps
Registration, Group Education and IEC Standard: Group Education Delivered with Correct Information	Lack of knowledge by health educators about the content of group education and no information on number of clients that attend the health education sessions.	Facility teams were supported to develop lists of talking points for group education to ensure that all key points were covered. Staff gained understanding of the importance of group education and key messages covered during these sessions. Notebooks were introduced to register all clients who attend group education, including noting presence of clients’ partners and parents/guardians who come to provide consent for minors.
Management Systems Standard: Relevant VMMC Policies, Guidelines and Standards are Available and Staff Are Aware of Them	Lack of key reference documents: VMMC Policy, HCT guidelines, QI framework and Strategic plan, STI syndromic management guidelines.	The teams identified a common place where reference documents would be kept and assigned a member of staff to take charge of the mini library. Sessions were organized in which the team would jointly peruse the documents to ensure they all have a common understanding of the key points in the guidelines.
Monitoring & Evaluation Standard: Availability of Relevant Tools in VMMC Clinic	Absence of standard M&E tools at national level.	USAID ASSIST worked with the MOH to expedite the process of developing standard M&E tools. Once draft tools were available, USAID ASSIST shared them with IPs and sites to use as they were being developed. This not only solved the challenge of poor M&E, but site teams were able to pilot and provided useful feedback which was used to refine the tools.

### Performance on Client-level Indicators

Figs 2–6 show time series data for five of the client-level performance indicators that the 30 sites tracked monthly.

#### Follow-up within 48 hours post-surgery

Fig 2 shows that the highest percentage of clients recorded turning up for review at 48 hours post-circumcision was 60 percent before the intervention in the four sites that had this data, but zero percent at one general hospital in February 2013. However, when teams were supported through monthly coaching visits by USAID ASSIST staff and representatives from the DHO in addressing this quality gap, significant improvement in client follow-up was observed.

#### Pre-surgical assessment of clients

Fig 3 shows that the percentage of clients who were assessed for STIs before circumcision was only 81 percent in January 2013. Three months after the intervention, significant improvement was observed in the proportion of clients assessed, and performance has stabilized at close to 100%.

#### Documentation of informed consent prior to circumcision

Fig 4 shows that the percentage of clients with signed consent forms was 80 percent at baseline. However, some sites like the general hospital shown in Fig 4 had consent rates as low as 0 percent, but raised their performance after the CQI activities.

#### Rate of moderate to severe adverse events

Documenting and measuring moderate to severe adverse events (AEs) is important in ensuring optimal outcomes. In January 2013, 18 sites reported on the medical circumcision procedure, but only nine (50 percent) had information documented related to the presence or absence of adverse events. By March 2014, all sites that reported on VMMC also reported on AEs. Fig 5 shows that at baseline the rate of adverse events was below 1% at the nine sites that had the data. After orienting staff on identification and grading of adverse events, the rate initially rose to 2% across all 30 sites and to 5.2% at one regional referral hospital, as sites improved their documentation of adverse events. Following efforts to actively reduce adverse events, the rate dropped below 1% across all sites and below 2% at the regional referral hospital for 10 months until April 2014, when it rose above 2%.

#### Circumcision under local anesthesia

At the time of baseline assessment in January to March 2013, not all clients were being circumcised under local anesthesia, which is the MOH norm for VMMC. Fig 6 shows that whereas the aggregate results from several sites showed use of local anesthesia at almost 100 percent, at some health units, like the Health Center (HC) IV shown in the Fig 6, providers still used general anesthesia and sedation. After orienting the staff on how to give local anesthesia and the non-compliant use of general anesthesia for VMMC, within one month of support, there was no reported use of general anesthesia for a period of one year.

## Discussion

This work sought to describe use of a continuous quality improvement approach to address quality gaps in the PEPFAR-supported VMMC program in Uganda. Whereas several assessments have been conducted to ascertain the safety and quality of VMMC [7,11,12] and adaptation of surgical efficiency models [13], to the best of our knowledge, little has been done to support VMMC teams to systematically address quality gaps at every single site as the service is scaled up. Among the 30 health units supported was a mobile van. We found that the performance of this health unit was comparable to the static health units supported by the same implementing partner.

From the baseline results, the worst performing area was M&E which was not surprising because there were previously no standard client data collection tools in the country. Each site and IP had improvised their own tools, most of which lacked key data elements. There were also no standard operating theater registers, and this greatly affected the M&E area. Part of the early intervention was to make uniform tools available to all sites. This was done by sharing the draft tools with sites. The partners supported each site team to print a few copies so that when a revised version of the tool became available, the old version was discarded. Teams were also provided with onsite orientation in use of the tools, and this greatly improved data quality. Some key quality indicators, such as documentation of consent and recording of type and grade of AEs, which had not previously been tracked, became feasible to measure, and teams were able to track their performance on these indicators and make improvements where necessary.

The second most challenging area at baseline was management systems because part of the standards required presence of functional improvement teams which were absent at most sites. The management systems area also required presence of VMMC work plans and adequate number of trained staff. These were absent in the majority of sites. This greatly affected implementation of the service: stock-outs were common because there was no plan for supplies, especially for the routine (i.e., not at outreach camps) circumcisions. A few sites did have work plans for outreach camp activities, but most of these were mainly budgets for staff allowances and lacked clear objectives of what they intended to accomplish.

Some sites did not have adequate number of trained staff, and at some sites, staff were performing roles for which they had not been trained or were not legally allowed to perform. For instance, there were many situations where nurses trained as assistant circumcisers were working as circumcisers, despite the law not permitting this. Working with IPs, training gaps were identified, and health facilities were supported to have more staff trained in specific roles and responsibilities.

During the assessments, scores were assigned to each standard assessed, and a total score for that area was obtained. At first, a scoring system was used to give feedback to teams about their performance, but USAID ASSIST staff noted that most team members did not appreciate the magnitude of the gap, and some were displeased with the scores. Hence, the idea of the dashboard was developed. The dashboard aroused their interest to critically interpret assessment findings. This motivated teams to work towards specific goals. Teams that were in the green were also encouraged to ensure that they maintained the good performance, as no team wished to be informed that their performance had declined, though such cases did occur. The effect of the dashboard was to spur a sense of friendly competition among sites, since all wished to be seen as good performers, in the “green.”

Whereas teams have managed to improve performance of individual standards and the dashboard is “greener,” there is still more to do. Sites are being supported to ensure that they attain a “green” of 100 percent and not just scoring 80 percent. There is also a need to ensure that sites maintain the gains achieved. Current support is focused on sustaining achievements reached at the 30 sites and transferring successful changes to other sites. Plans are underway to scale up CQI support to all VMMC sites through building a large pool of improvement coaches at district level to support the spread of best practices.

In addition to raising compliance with the 53 MOH standards, teams were also supported to improve client-level indicators. We observed significant improvement in all client-level indicators except the rate of AEs. This may be due to the fact that a good number of teams put more efforts into identification and documentation of AEs. As shown in Fig 5, at baseline only nine teams were collecting data on AEs, but this number gradually increased to tally with the number of sites that reported circumcision. Therefore as this process improves, a *false* worsening of the rate of AEs is expected to occur. At baseline, only four of the 30 health units were collecting data on clients who return for follow-up within 48 hours of circumcision. This was mainly attributed to lack of awareness of the importance of follow-up, and further compounded by the information provided to clients: clients would be informed to return only if they had problems, otherwise they were told to remove the bandage themselves. During the baseline assessment, sites were informed about the importance of client follow-up after circumcision, and as a result, more sites took interest in this and started collecting performance data.

Using an example of one general hospital, Fig 2 shows some of the changes that this site tested and the impact these changes had on the percentage of clients who returned for follow-up. At the beginning of the improvement intervention, the team at the general hospital was informing circumcised clients to return only if they had a problem, and only 1 percent of the clients would come back for follow-up. During coaching visits, the site was supported to form an improvement team and mentored on the importance of client follow-up. This led to a modest improvement from 1 percent in April 2013 to 32 percent in May 2013. The team noted that after changing the information given to clients, the number returning and attended to was high, but their records were reflecting poor performance. The team identified a problem where clients were returning for follow-up and attended to, but this information was not recorded. It was not until the team improved documentation to ensure all clients who returned for follow-up were recorded that the actual number of clients returning was known. When documentation was improved, it showed that the site had significantly improved in client follow-up. Improvement in documentation was combined with informing the clients to come for bandage removal—a reason clients found very convincing.

Prior to the intervention, about 20 percent of clients would get circumcised without being assessed for STIs (Fig 3). This had the potential to lead to AEs. Sites were supported to ensure that all clients are assessed for STIs. Some of the changes tested are shown in Fig 3 using the example of one health center level IV (HC IV). At the HC IV, less than 5 percent of the clients circumcised were being assessed prior to the surgical procedure. This was partly due to staff not being aware about the importance of STI assessment and also due to poor documentation, in which case clients would be assessed but no documentation would be done. A continuing medical education (CME) session was conducted in which the importance of STI assessment and documentation was discussed; this led to better performance. However, it was not until circumcisers were assigned the role of ensuring all records were fully completed that significant improvement was observed.

*“All improvement will require change*, *but not all change will result in improvement”* [18]. Not all the changes that sites tested led to better VMMC performance. For example, some sites tried referring clients to health facilities closer to where they lived for review after circumcision. However, this did not yield the expected results as the health workers at the referral facilities were not willing to complete the work of the site that conducted the circumcision. In most cases, these were small health facilities with few staff that lacked enough supplies, like gloves, and thus found it difficult to attend to high numbers of circumcision clients.

While improvement in VMMC quality measure has occurred in the 30 sites, it has not been without challenges. Improvement teams often get disorganized when there is change in staffing, and this slows down the improvement activities. Secondly, the activity started at a time when most of the IPs had already developed their annual work plans, which did not include this activity. It therefore became challenging to have them on board for all the joint activities we implemented. Related to this was the fact that some of the identified gaps were beyond the means of the QI team and the DHO—they required the intervention of the partner, yet some IPs never had budgets for these interventions, such as training service providers in VMMC.

## Limitations

First, the study relied on secondary data collected by the site teams for the client-level indicators. Only a few of the sites were collecting all the required data at baseline; many sites had some of the data, but it was incomplete. If the actual performance of those sites that did not have documentation was higher than that of sites that did have data, then the baseline may have underestimated overall performance.

The assessment of performance against the quality standards was done at specific points in time. This cross-sectional data only indicates the prevailing circumstances at the time of the assessment and may not reflect day-to-day compliance with the standards. We tried to overcome this by conducting complete assessments on a quarterly basis and mini-assessments of problematic standards during coaching visits.

Quality improvement methods are mainly aimed at addressing system gaps. The changes tested that led to improvement may not necessarily lead to improvement elsewhere without being adapted in the new setting. Thus, the changes found by these sites to yield better performance, such as those summarized in Table 4, may not yield the same results in other sites.

## Conclusions

The experience of the Uganda VMMC improvement pilot program shows that it is possible to address the quality of medical male circumcision using a CQI approach. However, broad consultation with stakeholders before starting implementation is vital.

Plans to implement VMMC CQI should allow flexibility and adaptability to different contexts across sites, districts and regions. We strongly recommend that VMMC programs take into consideration quality improvement interventions from the inception of program design.

Use of simple but very informative tools to understand improvement is key. The dashboard is one such tool that is easily understood by most stakeholders.

Facility-led QI processes are effective in quickly addressing quality gaps because they are driven by local managers and stakeholders. Health workers have solutions to most of the gaps they have in service delivery. They only need guidance on implementation of improvement work.
